# A Preoperative Clinical Risk Score Including C-Reactive Protein Predicts Histological Tumor Characteristics and Patient Survival after Surgery for Sporadic Non-Functional Pancreatic Neuroendocrine Neoplasms: An International Multicenter Cohort Study †

**DOI:** 10.3390/cancers12051235

**Published:** 2020-05-14

**Authors:** Florian Primavesi, Valentina Andreasi, Frederik J.H. Hoogwater, Stefano Partelli, Dominik Wiese, Charlotte Heidsma, Benno Cardini, Eckhard Klieser, Katharina Marsoner, Uwe Fröschl, Sabine Thalhammer, Ines Fischer, Georg Göbel, Andreas Hauer, Tobias Kiesslich, Philipp Ellmerer, Reinhold Klug, Daniel Neureiter, Helwig Wundsam, Franz Sellner, Peter Kornprat, Reinhold Függer, Dietmar Öfner, Elisabeth J.M. Nieveen van Dijkum, Detlef K. Bartsch, Ruben H.J. de Kleine, Massimo Falconi, Stefan Stättner

**Affiliations:** 1 Department of Visceral, Transplant and Thoracic Surgery, Medical University of Innsbruck, 6020 Innsbruck, Austria; florian.primavesi@tirol-kliniken.at (F.P.); benno.cardini@i-med.ac.at (B.C.); dietmar.oefner@i-med.ac.at (D.Ö.); 2 Pancreatic Surgery, Università Vita-Salute, IRCCS Hospital San Raffaele, 20132 Milan, Italy; andreasi.valentina@hsr.it (V.A.); partelli.stefano@hsr.it (S.P.); falconi.massimo@hsr.it (M.F.); 3 Department of Surgery, University Medical Center Groningen, University of Groningen, 9713 GZ Groningen, The Netherlands; f.j.h.hoogwater@umcg.nl (F.J.H.H.); r.de.kleine@umcg.nl (R.H.J.d.K.); 4 Department of Visceral, Thoracic, and Vascular Surgery, University Hospital Marburg, 35043 Marburg, Germany; wiesed@med.uni-marburg.de (D.W.); bartsch@med.uni-marburg.de (D.K.B.); 5 Department of Surgery, Cancer Center Amsterdam, Amsterdam UMC, University of Amsterdam, 1105 AZ Amsterdam, The Netherlands; c.m.heidsma@amsterdamumc.nl (C.H.); e.j.nieveenvandijkum@amsterdamumc.nl (E.J.M.N.v.D.); 6 Institute of Pathology, Paracelsus Medical University, 5020 Salzburg, Austria; e.klieser@salk.at (E.K.); d.neureiter@salk.at (D.N.); 7 Department of Surgery, Medical University Graz, 8036 Graz, Austria; katharina.marsoner@kages.at (K.M.); peter.kornprat@medunigraz.at (P.K.); 8 Department of Surgery, Ordensklinikum, 4010 Linz, Austria; uwe.froeschl@ordensklinikum.at (U.F.); ines.fischer@ordensklinikum.at (I.F.); helwig.wundsam@ordensklinikum.at (H.W.); reinhold.fuegger@ordensklinikum.at (R.F.); 9 Department of Surgery, Kaiser Franz Josef Hospital, 1100 Vienna, Austria; sabine.thalhammer@wienkav.at (S.T.); sellner.franz@aon.at (F.S.); 10 Department of Medical Statistics, Informatics and Health Economics, Medical University of Innsbruck, 6020 Innsbruck, Austria; georg.goebel@i-med.ac.at; 11 Department of Surgery, General Hospital Horn, 3580 Horn, Austria; andreas.hauer@horn.lknoe.at (A.H.); reinhold.klug@horn.lknoe.at (R.K.); 12 Institute of Physiology and Pathophysiology, Paracelsus Medical University, 5020 Salzburg, Austria; t.kiesslich@salk.at; 13 Department of Neurology, Medical University of Innsbruck, 6020 Innsbruck, Austria; philipp.ellmerer@tirol-kliniken.at

**Keywords:** pancreatic neuroendocrine tumors, C-reactive protein, risk score, neuroendocrine neoplasms, pancreas, surgery, survival

## Abstract

*Background:* Oncological survival after resection of pancreatic neuroendocrine neoplasms (panNEN) is highly variable depending on various factors. Risk stratification with preoperatively available parameters could guide decision-making in multidisciplinary treatment concepts. C-reactive Protein (CRP) is linked to inferior survival in several malignancies. This study assesses CRP within a novel risk score predicting histology and outcome after surgery for sporadic non-functional panNENs. *Methods:* A retrospective multicenter study with national exploration and international validation. CRP and other factors associated with overall survival (OS) were evaluated by multivariable cox-regression to create a clinical risk score (CRS). Predictive values regarding OS, disease-specific survival (DSS), and recurrence-free survival (RFS) were assessed by time-dependent receiver-operating characteristics. *Results:* Overall, 364 patients were included. Median CRP was significantly higher in patients >60 years, G3, and large tumors. In multivariable analysis, CRP was the strongest preoperative factor for OS in both cohorts. In the combined cohort, CRP (cut-off ≥0.2 mg/dL; hazard-ratio (HR):3.87), metastases (HR:2.80), and primary tumor size ≥3.0 cm (HR:1.83) showed a significant association with OS. A CRS incorporating these variables was associated with postoperative histological grading, T category, nodal positivity, and 90-day morbidity/mortality. Time-dependent area-under-the-curve at 60 months for OS, DSS, and RFS was 69%, 77%, and 67%, respectively (all *p* < 0.001), and the inclusion of grading further improved the predictive potential (75%, 84%, and 78%, respectively). *Conclusions:* CRP is a significant marker of unfavorable oncological characteristics in panNENs. The proposed internationally validated CRS predicts histological features and patient survival.

## 1. Introduction

Pancreatic neuroendocrine neoplasms (panNENs) represent heterogeneous and rare tumors despite a rising incidence [1]. Treatment and outcome are determined by underlying genetic syndromes (e.g., multiple endocrine neoplasia), functional activity (e.g., insulinoma), symptoms (flushing, pain, jaundice), and histopathological variables like grading or lymph node status [2,3,4]. Accordingly, the therapeutic landscape comprises a variety of treatments, including somatostatin analogs, peptide receptor radionuclide therapy (PRRT), chemotherapy, surgery, and ablation [5]. However, in most panNENs, surgery remains the cornerstone of curative intent therapy in resectable disease [6].

Several risk-scoring systems have been proposed [7,8,9], and most of them are composed of factors not reliably available before surgery, e.g., lymph node positivity or microvascular/perineural invasion. Additionally, tumor grading is often included, but assessment by means of preoperative biopsy with the Ki-67 proliferative index may be challenging due to insufficient samples or intratumoral heterogeneity [10,11,12]. Thus, for the prediction of postoperative morbidity, histological features, and estimated overall, disease-specific, and recurrence-free survival (OS, DSS, and RFS), a risk score with preoperative ideally non-invasively available factors is highly desirable for guidance of potential (neo-)adjuvant therapeutic concepts, especially in high-risk individuals [13,14].

The systemic inflammatory status affects tumor growth, metastatic behavior, and the host response in numerous malignancies, including pancreatic and esophageal carcinoma, colorectal cancer, and gastroenteropancreatic neuroendocrine tumors. C-reactive protein (CRP) has been assessed as a marker of outcome in a plethora of tumor entities [15,16,17,18,19,20]. Furthermore, its potential to promote malignancy in panNENs has been shown in an in vitro study [21], and an association with long-term patient outcomes was confirmed in a retrospective single-center analysis [22]. However, conclusions from the latter study are limited since it included a rather heterogeneous cohort and lacked external validation or correlation of CRP with postoperative complications. 

The present work utilized national multicenter data (Austrian Society of Surgical Oncology/ASSO cohort [2]) validated by an international multicenter cohort (IMC) to evaluate the association of preoperative CRP with postoperative histology, surgical short-term, and oncological long-term outcomes after curative intent resection of sporadic non-functional (NF) panNENs. Furthermore, a novel predictive risk score based on purely preoperatively available factors is presented.

## 2. Results

### 2.1. Patient Characteristics, Procedures, and Outcomes

In summary, 364 patients undergoing curative intent pancreatic resection were included, 160 from the ASSO cohort and 204 from the IMC. In total, 104 patients (28.6%) underwent pancreaticoduodenectomy, 190 (52.2%) distal resection, 52 (14.3%) enucleation, and 18 (4.9%) total pancreatectomy. Here, 90-day mortality was 2.7%, and overall and severe morbidity were 58% and 10.2%, respectively. Overall morbidity (*p* = 0.773) and severe morbidity (*p* = 0.854) were not associated with OS. Table 1 presents the characteristics of patients and tumors as well as the outcomes according to the two cohorts. The national cohort was comprised of patients with a higher median age, and preoperative CRP, as well as significantly more advanced panNENs in terms of grading, T categories, and the presence of metastases.

The estimated 5-year and 10-year OS was 84.7% and 66.2% in the whole cohort (median: 321.9 months; 95%CI not computable), with 80.1% and 57.0% in the ASSO and 89.5% and 76.4% in the IMC cohort (*p* = 0.069). The 5-year and 10-year DSS was 91.2% and 79.2% in the whole cohort (median not reached), with 87.0% and 76.9% in the ASSO and 96.5% and 90.7% in the IMC cohort (*p* = 0.037). The estimated 5-year and 10-year RFS was 72.2% and 59.2% in the whole cohort (median not reached), with no significant difference between the ASSO and IMC cohort (70.8% and 56.9% compared to 72.7% and 60.9%; *p* = 0.358).

### 2.2. Association of Increased CRP with Other Factors and Outcome

**Figure 1:**
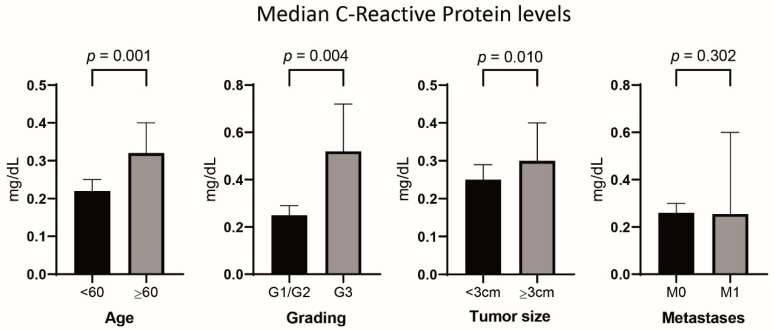
C-reactive protein levels in the whole cohort stratified by patients’ age, grading, tumor size, and presence of metastases (bar charts indicate median values and 95% confidence intervals).

The median CRP levels were significantly higher in patients ≥60 years and in cases with tumor size ≥3 cm or with G3 grading (Figure 1). Optimal cut-offs in regard to association with OS and DSS were calculated at ≥0.2 mg/dL for high versus low CRP and ≥3.0 cm for small versus large tumor size. Patients with CRP values ≥0.2 mg/dL (*n* = 226 of 364) revealed a significantly increased median age as well as a higher frequency of symptoms other than pain (see Table S1, Supplemental Digital Content). Additionally, tumor characteristics associated with an aggressive biology, such as G3 grading (*p* = 0.048) and size ≥3 cm (*p* = 0.090), were more common in the high-CRP group.

### 2.3. Analysis of Preoperative Factors and Survival

**Figure 2:**
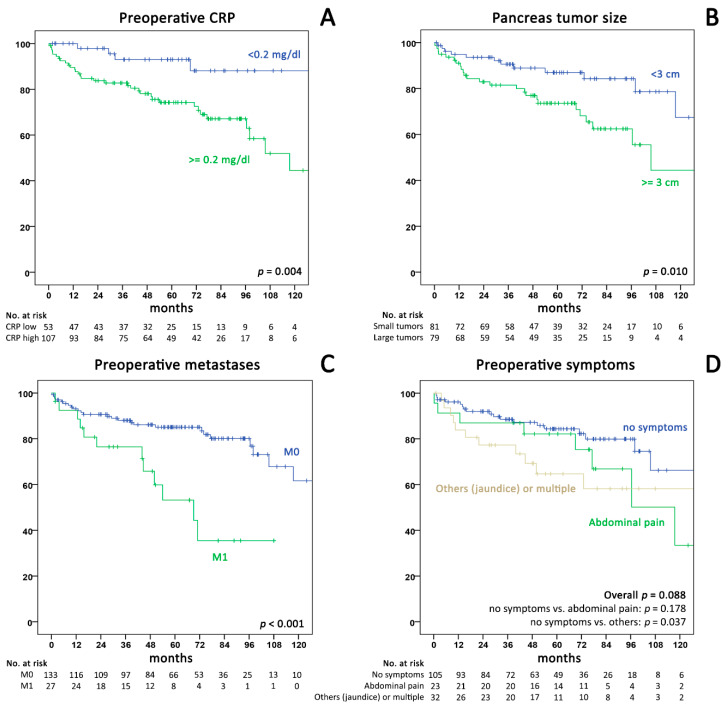
Kaplan–Meier curves for overall survival in the national ASSO cohort stratified by (**A**) preoperative CRP (cut-off 0.2 mg/dL; *p* = 0.004), (**B**) tumor size (cut-off 3 cm; *p* = 0.010), (**C**) metastases (*p* < 0.001), (**D**) symptoms (no symptoms versus pain *p* = 0.178; no symptoms versus other/multiple symptoms *p* = 0.037). CRP = C-reactive protein.

Univariable analysis of preoperative factors and OS after surgery in the ASSO cohort (Figure 2 and Table 2) showed a significant association with CRP ≥0.2 mg/dL (HR 4.13; 95%CI 1.46–11.7), metastases (HR: 3.32; 95%CI 1.64–6.71), tumor-size ≥3 cm (HR 2.41; 95%CI 1.21–4.82), symptoms other than pain (HR 2.18; 95%CI 1.03–4.62), and age (HR: 1.59 per 10 years; 95%CI 1.18–2.13). Multivariable analysis confirmed the independent association of CRP with decreased OS in the ASSO (HR 2.98) and the IMC cohort (HR 6.30), alongside the presence of metastases (HR 2.57 in the ASSO cohort) and age (HR 1.6 in both cohorts; all *p* < 0.05). In the combined analysis of all 364 patients, CRP ≥0.2 mg/dL (HR 3.87; 95%CI 1.65–9.07; *p* = 0.002), metastases (HR 2.80; 1.49–5.25; *p* = 0.001), tumor size ≥3 cm (HR 1.83; 95%CI 1.05–3.16; *p* = 0.034), and age (HR 1.56 per 10 years; 95%CI 1.24–1.97; *p* < 0.001) remained significant factors for OS. Due to the limited availability of preoperative biopsies with grading in our cohort, we did not include this variable into the initial regression analysis.

### 2.4. Association of Preoperative CRP with OS, DSS, and RFS

To investigate the underlying causal coherences of CRP and decreased OS, we further investigated factors associated with recurrence. Relapse of panNEN was significantly associated with decreased OS (median 117.5 months vs. median not reached; 5-year OS: 67.7% vs. 91.9%; *p* < 0.001). Additionally, CRP ≥0.2 mg/dL was linked to pan-NEN-related deaths (both median not reached; 5-year DSS: 87.7% vs. 98% and 10-year DSS: 78.2% vs. 98%; *p* = 0.002) However, CRP ≥0.2 mg/dL was not associated with inferior RFS (both median not reached; 5-year RFS: 72.2% vs. 70.4%; *p* = 0.893) or a significantly increased recurrence rate (25.6% vs. 21.2%; *p* = 0.343) We therefore hypothesized that recurrence in high-CRP cases might not occur earlier or more often but may be associated with more aggressive behavior of tumor relapse and impaired host-defensive abilities. Patients with increased CRP were found to have more multisite or extra-pancreatic/extra-hepatic recurrence (43.9% vs. 34.5%), although this was not statistically significant (*p* = 0.403). Additionally, survival after recurrence was used as a surrogate to determine if relapse more frequently leads to patient death in cases with high baseline CRP. In patients with recurrence (*n* = 86), median OS from the time point of recurrence was markedly different between patients with high CRP (*n* = 57) versus low CRP (*n* = 29): 29.3 months (95%CI 8.5–50.0) versus 62.2 months (95%CI 0.0–132.8; *p* = 0.004). This also applied to panNEN-related death rates after recurrence with a 5-year DSS of 40.3% compared to 88% (median 42.5 months, 95%CI 27.4–57.6 vs. not median reached; *p* = 0.003).

### 2.5. Development of a Clinical Risk Score Including CRP

To create a preoperative clinical risk score (CRS) allowing an estimate of postoperative patient survival, we combined three factors most strongly associated with OS in the multivariable model (CRP ≥0.2 mg/dL, presence of metastases, and tumor size ≥3 cm). Each factor was assigned one point, resulting in four scoring groups with increasing risk (CRS:0 to CRS:3). The characteristics of patients in each group in the combined panNEN cohort are presented in Table 3. A higher CRS was associated with histologically aggressive characteristics, such as G2/G3 grading (*p* < 0.001), T3/T4 category (*p* < 0.001), and lymph node positivity (*p* = 0.002). Moreover, the 90-day overall morbidity (*p* = 0.006) and mortality (*p* = 0.004) were increased in CRS-high patients, reflecting the technical challenges of resection in advanced disease cases. Ultimately, the 5-year and 10-year OS, DSS, and RFS were all markedly reduced in these patients (*p* < 0.001), even after removing patients with 90-day mortality (all *p* < 0.001).

In line with the abovementioned different baseline characteristics of the ASSO and IMC cohorts (Table 1), 43.1% of patients presented with a CRS:2 or CRS:3 in the ASSO cohort, while only 27.4% were graded similarly in the IMC cohort (*p* = 0.004).

### 2.6. Oncological Outcome According to the Clinical Risk Score

**Figure 3:**
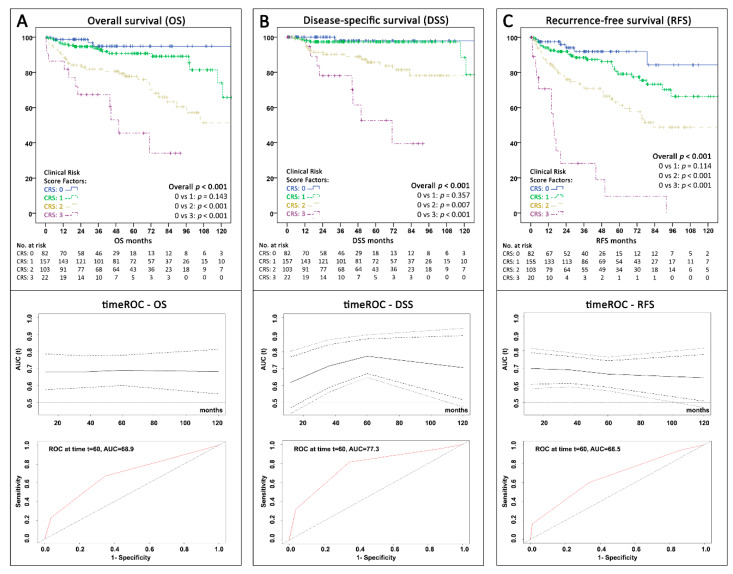
Kaplan–Meier curves with time-dependent receiver operating characteristics (ROC) (+95% confidence intervals) over 120 months and area under the curve (AUC) at 60 months showing the survival and predictive value according to the proposed clinical risk score with three factors (presence of metastases, CRP ≥0.2 mg/dL, and primary tumor-size ≥3 cm) for the whole cohort (*n* = 364) (**A**) Overall survival (**B**) Disease-specific survival (death due to panNEN progression) (**C**) Recurrence-free survival. AUC = Area under the curve; CRS = Clinical risk score; ROC = receiver operating characteristics; t60 = timepoint 60 months; timeROC = time-dependent receiver operating characteristics.

From CRS:0 to CRS:3, each additional risk factor resulted in an increased probability of death (OS) during follow-up with an HR of 2.62 (95%CI 1.91–3.60; *p* < 0.001) and panNEN-related death (DSS) with an HR of 3.53 (95%CI 2.20–5.68; *p* < 0.001; Cox-proportional hazards regression). Figure 3 depicts the Kaplan–Meier curves for OS, DSS, and RFS stratified by CRS groups in the combined cohort. The score significantly predicted OS (Figure 3A; overall *p* < 0.001), although with limited discrimination between low-risk subgroups (*p* = 0.143). Time-dependent ROC analysis revealed an AUC of 68.9 (95%CI 61.5–76.4) to predict OS at 60 months. The score was significantly associated with DSS (overall *p* < 0.001; Figure 3B), again with limited discrimination between CRS:0 and CRS:1 patients (*p* = 0.357) but with a markedly higher 60-month AUC of 77.3 (95%CI 67.2–87.5). Finally, although not initially created for recurrence prediction, the CRS showed a solid value for RFS estimation with an AUC of 66.5 (95%CI 58.9–74.2) and significant discrimination (overall *p* < 0.001; Figure 3C). Despite the differences in characteristics between the ASSO and IMC cohort, the CRS showed a significant association with OS, DSS, and RFS in both cohorts independently (all overall *p* ≤ 0.005; see Figure S1, Supplemental Digital Content).

### 2.7. Implementation of Grading in the Proposed Risk Score

**Figure 4:**
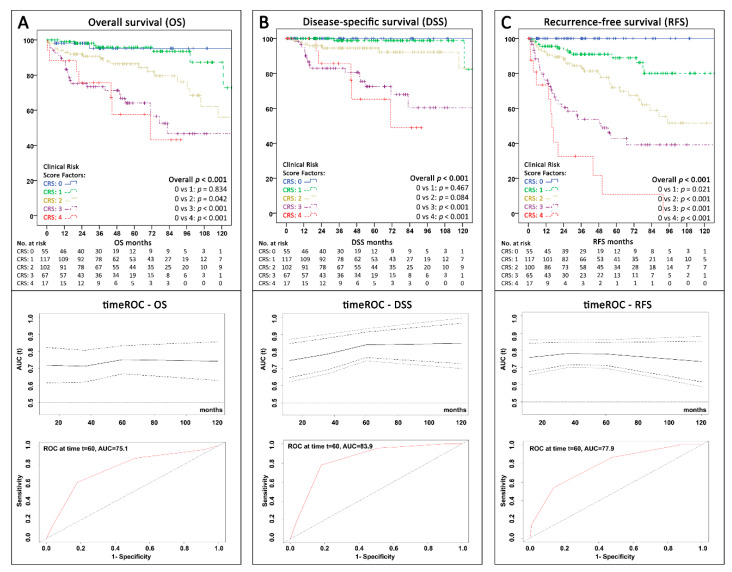
Kaplan–Meier curves with time-dependent ROC (+95% CI) over 120 months and AUC at 60 months showing the survival and predictive value according to the extended version of the proposed clinical risk score with 4 factors (World Health Organization (WHO) grading G1 vs. G2/G3, presence of metastases, CRP ≥0.2 mg/dL, and primary tumor-size ≥3 cm) for the whole cohort (n = 358; grading missing = 6) (**A**) Overall survival (**B**) Disease-specific survival (death due to panNEN progression) (**C**) Recurrence-free survival. AUC = Area under the curve; CRS = Clinical risk score; ROC = receiver operating characteristics; t60 = timepoint 60 months; timeROC = time-dependent receiver operating characteristics

According to the plethora of evidence on the association of grading with panNEN outcomes, we further analyzed the value of including grading in the proposed CRS. In our cohort, preoperative biopsies were performed in 134 of 364 patients (36.8%), and 38 of these were reported as insufficient tissue material for Ki67 staining or showed inconclusive histology. There was no significant difference in the rate of availability of preoperative grading between the ASSO and IMC cohort (23.1% vs. 28.9%; *p* = 0.213). Of the 96 patients with positive biopsy and available grading, 49 were preoperatively graded as G1, 37 as G2, and 9 as G3. Notably, their final surgical specimen grading was only concordant in 66 cases (69.5%). In view of the long study period, we assessed, whether the latest developments in biopsy and imaging techniques recently led to increased utilization of preoperative tissue acquisition resulting in correct grading in a higher percentage of patients. Intriguingly, within the last three years of our inclusion period (2016–2018), the rate of patients with representative preoperative samples (47.3%) as well as correct grading (32.3% of all patients) was significantly higher than in the years before (1990–2015; 19.2% and 13.7%). Accordingly, and in order to maximize statistical power, we therefore used the surgical specimen grading as a surrogate for preoperative grading. When resection specimen grading was included as an additional factor in multivariable analysis, it showed an independent significant association with OS (HR 2.09; 95%CI 1.17–3.74; *p* = 0.013; Table S2, Supplemental Digital Content). Amending the proposed CRS by adding grading (presence of G2 or G3 versus G1 as another point in the risk score) resulted in a further significant improvement of the predictive ROC values throughout all different types of survival analysis (see Figure 4A–C).

### 2.8. Applicability of the CRS in the Presence of Positive Functional Imaging

Besides recently increased rates of preoperative biopsy, more frequent application of functional somatostatin-receptor (SSR) imaging (e.g., octreotide scan or DOTATOC-positron emission tomography computed tomography (PET-CT)) has also substantially changed preoperative assessment for panNENs lately. Functional imaging was performed in 217 of 364 patients (59.6%), and panNEN-typical tracer uptake was more common with DOTATOC-PET-CT (129 of 138; 93.5%) compared to the octreotide scan (61 of 79; 77.2%; *p* < 0.001). Within the most recent years (2016–2018), 82.8% of all patients received preoperative functional imaging compared to 51.7% in the years before (1990–2015; *p* < 0.001). More specifically, this was caused by increased application of DOTATOC-PET-CT (78.5% vs. 24%) with concurrent reduced utilization of octreotide scans (4.3% vs. 27.7%; *p* < 0.001). This ultimately also resulted in increased SSR imaging positivity recently (94.8% vs. 83.6%; *p* = 0.018).

In the whole cohort, when selecting SSR imaging-positive patients only, both the three-risk factor CRS and modified four-risk factor CRS remained highly significantly associated with overall, disease-specific, and recurrence-free survival (overall *p*-values for 3-factor CRS: OS 0.002, DSS <0.001, RFS <0.001; for 4-factor CRS: OS 0.001, DSS <0.001, RFS <0.001).

Taken together with the findings described above on grading, these results confirm that our CRS is specifically applicable in a state-of-the-art setting of panNEN assessment in specialized centers with routine availability of accurate preoperative grading and SSR imaging.

All patient data used for this study are provided in anonymized form in a supplement excel file in the Supplementary Material (Table S3).

## 3. Discussion

This international study assessed the value of preoperative CRP to predict histological features as well as postoperative outcome after resection of sporadic NF panNENs. Increased CRP is associated with patient factors (age, symptoms), as well as tumor characteristics (grading, tumor size) and OS. Analysis of DSS, type of recurrence, and survival after relapse suggests that CRP is linked to biologically more aggressive panNENs. We furthermore showed that a CRS incorporating CRP ≥0.2 mg/dL, tumor-size ≥3 cm, and the presence of metastases enables preoperative stratification by the prediction of postoperative 90-day morbidity and mortality, unfavorable oncological parameters (grading, T category, and lymph node involvement), rate of recurrence, and poor long-term survival. Importantly, these findings were not only explored on a national multicenter level but also independently validated in four European large-scale units for pancreatic surgery (including three ENETS centers of excellence). Further inclusion of grading in a modified scoring version and evaluation of both CRSs in SSR imaging-positive patients suggests the usefulness in state-of-the-art panNEN management and a clinically relevant potential for stratification in future studies on (neo)adjuvant therapies or personalized follow-up.

Previous risk scores for panNEN rely on postoperatively assessed factors or include biomarkers without broad availability, limiting clinical applicability for preoperative stratification. Genc et al. and Zaidi et al. recently presented scoring systems to predict recurrence for G1/G2 NF-panNENs based on mostly postoperatively available factors [7,9]. Their scores mainly aid in decision-making regarding an indication for adjuvant treatment and intervals of follow-up. An analysis comparable to our work assessed chromogranin A (CgA) regarding recurrence after panNEN surgery [8], combining CgA (>5× upper normal limit) with tumor size (≥4 cm) and G2/G3 grading to design a preoperative risk score. Despite a 95% negative predictive value for recurrence in patients with zero points, the scoring system is limited by the restricted availability of CgA in some centers and possible falsely elevated CgA levels [23]. To overcome parts of these limitations, vasostatin-1 (VS-1), a cleavage fragment of CgA, has recently been explored as a prognostic biomarker, but VS-1 assessment is also not generally available and prospective validation is pending [24,25].

We therefore primarily focused on risk stratification prior to surgical resection through the inclusion of a simple cost-effective laboratory test (CRP) and established imaging. The novel CRS not only represents a valuable tool to inform patients on their perioperative risk and potential long-term outcome but also seems potentially useful to stratify high-risk panNEN patients for neoadjuvant PRRT, a therapeutic concept that is soon to be investigated in a prospective multicenter setting in Italy. In the future, inflammatory markers may also guide treatment strategies regarding immune therapy in panNEN patients [21,26,27,28].

The role of the systemic inflammatory response has been extensively characterized in a variety of different malignancies [29]. Regarding operable cancers, >300 studies on different inflammatory parameters and scores have recently been investigated in a meta-analysis [30]. In summary, the systemic inflammatory response has independent prognostic value for OS and cancer-specific survival across many tumor types. This enormous evidence calls for routine perioperative assessment of the host inflammatory status. In panNENs, the existing evidence is far less extensive, where the neutrophil-lymphocyte ratio (NLR) was assessed in only two single-center studies [31,32], confirming an association with disease progression and OS. CRP has been examined in one retrospective single-center cohort including a variety of different panNENs and treatments [22], and the authors found a CRP cutoff of ≥0.5 mg/dL optimally predicting OS. None of these studies was designed to draw valid conclusions regarding prognosis after surgery of sporadic NF panNENs.

In our study, the ideal cut-off for CRP to predict OS was considerably low with ≥0.2 mg/dL. Although this lies well below the upper normal range for detecting inflammation as defined by most laboratories around Europe (usually 0.7 mg/dL), this calculated value showed the highest significance in discriminating survival curves for OS in our analysis. Many initial studies on inflammation and cancer used a cut-off of 1.0 mg/dL, for example, as proposed in the original Glasgow prognostic score (GPS) [33]. However, more recently, the increased application of high-sensitivity CRP detection assays and lower cut-offs (usually around 0.3 mg/dL) within a “high-sensitivity” GPS has been shown to significantly improve patient risk stratification in a number of studies, e.g., on gastric cancer, lung cancer, and hepatocellular carcinoma [30,34,35,36].

The pathophysiological role of CRP as an acute-phase protein has been extensively investigated [37], but the causal relationship between raised CRP and poor survival in cancer patients seems unclear. It remains debatable if an increased inflammatory status is a byproduct of tumorigenesis, a reflection of the host responding to more aggressive tumor behavior, or a sign of an impaired ability of the host to control further tumor spread. It has been shown that increasing CRP during (healthy) ageing is a matter of fact [38]. In the elderly, elevated CRP is linked to poor physical status and cognitive performance, and individuals with increased inflammatory markers encounter an at least two times higher cancer incidence compared to those with normal values [39]. Additionally, chronically increased CRP is associated with disease severity in a number of comorbidities, such as cardiovascular disease or cirrhosis [39,40]. These medical conditions might also limit systemic or local treatment options in cancer patients with progressive recurrent disease after curative intent resection, and hereby CRP may also play an indirect role in poor cancer survival.

In our study, CRP was associated with tumor size and grading, suggesting a systemic measurable effect of pronounced host–tumor interactions. Schimmack et al. confirmed this through experimental studies in panNEN cell lines and patient tissue samples. Their results suggested a positive feedback mechanism between CRP and interleukin-6 (IL-6) in monocytes/macrophages maintaining systemic inflammation, leading to activation of the IL-6/AKT/signal transducer and activator of transcription protein 3 (STAT3)/CRP axis, which further promotes invasion and metastases [21,41,42]. Interestingly, in our cohort, although increased CRP did not correlate with decreased RFS, these patients showed more advanced recurrences and a significantly diminished OS and DSS. Additionally, in the case of recurrence, our patients with CRP ≥0.2 mg/dL experienced dramatically reduced OS in the further course of the disease.

The present works’ main limitation lies in its retrospective nature, making it difficult to draw high-level evidence-based conclusions. While prospective validation of our CRS would certainly be highly desirable, panNEN represents a rare and heterogeneous entity with a comparatively long survival, with possible changes in management over time. These inherent restrictions have particularly become evident during retrospective evaluation of histological grading and imaging. Late during the study inclusion period, in 2017, the World Health Organization (WHO) classification of panNENs was updated [43], introducing a differentiation between two sub-groups within G3 panNENs (G3 neuroendocrine tumour/NET vs. G3 neuroendocrine carcinoma/NEC). Histological subclassification of G3 panNENs was not routinely assessed in our cohort in the previous years and therefore our results should be interpreted with caution, especially regarding a potential influence of the G3 NEC subtype affecting preoperative CRP, recommended treatment options, and patient outcome. Furthermore, preoperative SSR imaging application and its diagnostic accuracy changed over different time periods as shown in the results. Interestingly enough, these diagnostics, nowadays considered to be standard of care according to guidelines, have only very recently been routinely implemented even in well-established international large units. Therefore, confirmation of our very specific but clinically relevant and intriguing findings will require a large-scale prospective validation approach with standardized pathological and imaging assessment. However, incorporating CRP and our risk score in treatment algorithms could substantially change clinical strategies for resectable sporadic NF panNENs.

## 4. Materials and Methods

### 4.1. Patient Cohort

This retrospective analysis was based on a national and international multicenter cohort. As previously described [2], the panNEN study group of the Austrian Society of Surgical Oncology (ASSO) collected the data of patients undergoing pancreatic resection in 6 Austrian centers. The international multicenter cohort (IMC) consists of four high-volume units, including three ENETS centers of excellence (IRCCS San Raffaele Hospital, Milan, Italy; University Hospital Marburg, Germany; Academic Medical Center Amsterdam, Netherlands; University Medical Center Groningen, Netherlands). Individual centers were free to choose their inclusion time period depending on data availability. Accordingly, reported patients underwent index pancreatic resection between 1998–2017 in the ASSO cohort and between 1990–2018 in the IMC.

### 4.2. Inclusion Criteria and Collected Variables

Inclusion criteria covered curative intent pancreatic resections for sporadic non-functional (NF) panNEN with postoperative histological diagnosis and availability of preoperative CRP (last value within 7 days before resection). Cases with simultaneous metastases were included only when a curative intent strategy for all lesions was performed (single- or multi-stage resection/ablation). Palliative debulking procedures were excluded, as were patients with simultaneous pancreatic adenocarcinoma. Postoperative 90-day complications were graded according the Clavien–Dindo-classification (severe: grade >3a) [44,45], pancreatic fistula according the ISGPF-2005 classification [46]. Here, 90-day mortality was validated through national registries in Austrian hospitals [47]. Tumor variables included intrapancreatic location, presence/location of metastases (assessed by cross-sectional imaging), and grading according the 2010 World Health Organization (WHO) classification (morphological mitotic rate (<2 in 10 high-power fields (HPFs): G1, 2-20/HPF: G2, and >20/HPF: G3) or proliferative activity by Ki-67-positive cells (G1, G2, and G3: ≤2%, 3–20%, >20%)). Staging was based on the European Neuroendocrine Tumor Society (ENETS) classification [48]. Nodal status was assessed on surgical specimens. Resection margin status was R0 for complete resections without microscopic residual tumor at the margin or R1 (positive margins) or Rx (missing).

Preoperative CRP was measured in a routine laboratory from whole-blood obtained plasma with a latex particle-enhanced turbidimetric assay in all centers. Primary tumor size was assessed through preoperative cross-sectional imaging when available or as described by pathology in the surgical specimen. OS was defined as the time from the first panNEN surgery to death/last follow-up, DSS as the time to panNEN related-death/last follow-up (non-panNEN-related deaths were censored), and RFS as the time from completion of the initial curative-intent concept to recurrence/last follow-up.

Data fusion and analysis was performed at Medical University of Innsbruck by two consultant HPB surgeons (S.S. and F.P.), reviewing each dataset concerning integrity and plausibility. Ethics committee approval was obtained independently by each participating department according to local regulations; no individual informed consent was claimed due to the retrospective study design. The EC approval numbers of the coordinating centers are 415-EP/73/408-2014 (Ethikkommission Land Salzburg) and AN2013-0023/AN2016-0128 (Ethikkommission der Medizinischen Universität Innsbruck). The study is reported according to the STrengthening the Reporting of OBservational studies in Epidemiology (STROBE) guidelines (see STROBE Checklist, Supplemental Digital Content) [49].

### 4.3. Statistical Analysis

Patient and tumor characteristics are described as a median with an interquartile range (IQR); for categorical variables, numbers and proportions are displayed. After testing for normal distribution with the Shapiro–Wilk test, medians were compared by the Mann–Whitney-U test between two groups and by the Kruskal–Wallis test for >2 groups. CRP-level bar charts are presented as a median with 95% confidence intervals (95%CI). Optimal cut-offs for CRP and tumor size regarding OS and DSS were established by systematically evaluating different values and selecting those with the lowest *p*-value in the log-rank-test and -2log-likelihood in Cox-regression. Cox-proportional hazards regression modeling was performed for an estimation of hazard ratios (HRs) with 95% CI of factors associated with survival. Sensitivity analysis was conducted to detect multicollinearity before entering factors into the multivariable model. Kaplan–Meier survival curves were plotted and the log rank test was used to assess subgroup differences. Time-dependent receiver-operating characteristics (ROC) area-under-the-curve (AUC) analysis was conducted using the timeROC package in R (www.r-project.org). This created a time-dependent ROC curve from censored survival data to calculate the AUC for predictive factors at a given time point (e.g., 60 months), as described by Blanche et al. [50]. All other statistical analyses were performed with SPSS v21.0 (IBM Corp. Armonk, NY, USA). A two-tailed *p*-value <0.05 was considered significant throughout all analysis.

## 5. Conclusions

Preoperative CRP is associated with unfavorable patient and tumor characteristics and independently predicts disease-associated survival in resected sporadic non-functional panNENs. Our internationally validated clinical risk score, including CRP, primary tumor size, and metastases (plus grading if available), facilitates easy clinical applicability and allows for risk stratification, potential guidance in future (neo-)adjuvant treatment regimens, and individualized follow-up recommendations.

## Figures and Tables

**Table 1:** Comparison of the national Austrian Society of Surgical Oncology (ASSO) cohort and international multicenter cohort (IMC) of patients undergoing curative intent surgery for pancreatic neuroendocrine neoplasm (panNEN).

Characteristics	ASSO (*n* = 160)	IMC (*n* = 204)	*P*
Female gender	76 (47.5%)	87 (42.6%)	0.355
Age (years; median; IQR)	62.4 (53.2; 69.5)	60.0 (50.0; 67.0)	0.037
Preoperative symptoms			0.093
No symptoms	105 (65.6%)	134 (65.7%)	
Pain	23 (14.4%)	43 (21.1%)	
Others (including jaundice) or multiple	32 (20.0%)	27 (13.2%)	
Preoperative Bilirubin ≥ 4 mg/dL	7 (4.4%)	10 (4.9%)	0.813
Preoperative CRP (mg/dL; median; IQR)	0.30 (0.12; 0.69)	0.25 (0.19; 0.65)	0.037
Metastases present	27 (16.9%)	13 (6.4%)	0.001
Primary tumor size ≥3 cm	79 (49.4%)	84 (41.2%)	0.118
Tumor location			0.068
Head	55 (34%)	81 (39.7%)	
Body/Tail	96 (60.0%)	120 (58.8%)	
Multiple	9 (5.6%)	3 (1.5%)	
ENETS T-category			0.003
T1/T2	90 (56.3%)	145 (71.1%)	
T3/T4	70 (43.8%)	59 (28.9%)	
Nodal status			0.249
Nodal-negative	102 (63.8%)	114 (55.9%)	
Nodal-positive	40 (25.0%)	57 (27.9%)	
Nodal-status unknown (Nx)	18 (11.3%)	33 (16.2%)	
Tumor grading (missing: 6)			0.002
G1	66 (42.0%)	120 (59.7%)	
G2	71 (45.2%)	68 (33.8%)	
G3	20 (12.7%)	13 (6.5%)	
R0 resection margin (missing: 3)	141 (89.8%)	184 (90.2%)	0.903
90-day severe morbidity	20 (12.5%)	17 (8.3%)	0.192
90-day mortality	6 (3.8%)	4 (2.0%)	0.345
Follow-up for OS (months; median; IQR)	57.5 (28.3; 83.3)	38.2 (20.1; 73.2)	0.006
Death during follow-up	37 (23.1%)	27 (13.2%)	0.014
Estimated 5-yr/10-yr OS after resection *	80.1%/57%	89.5%/76.4%	0.069
Estimated 5-yr/10-yr DSS after resection *	87.0%/76.9%	96.5%/90.7%	0.037
Recurrence during follow-up (missing: 4)	43 (27.6%)	43 (21.1%)	0.153

* median not reached in the ASSO cohort, therefore 5/10-year survival is presented. ASSO = Austrian Society of Surgical Oncology; CRP = C-reactive protein; DSS = Disease-specific survival; ENETS = European Neuroendocrine Tumor Society; IMC = International Multicenter Cohort; IQR = Interquartile range; OS = Overall survival; yr = year.

**Table 2:** Univariable and multivariable analysis of preoperative factors associated with overall survival.

	Univariable Analysis		Multivariable Analysis					
Factor	ASSO Cohort (*n* = 160)		ASSO Cohort (*n* = 160)		IMC Cohort (*n* = 204)		Combined (*n* = 364)	
	HR (95%CI)	*p*	HR (95%CI)	*p*	HR (95%CI)	*p*	HR (95%CI)	*p*
Male sex	1.23 (0.64–2.35)	0.525	1.26 (0.65–2.44)	0.499	0.83 (0.37–1.86)	0.647	1.02 (0.62–1.69)	0.931
CRP (≥0.2 mg/dL)	4.13 (1.46–11.7)	0.007	2.98 (1.03–8.62)	0.044	6.30 (1.46–27.3)	0.014	3.87 (1.65–9.07)	0.002
TU size (≥3 cm)	2.41 (1.21–4.82)	0.013	1.58 (0.75–3.32)	0.231	2.29 (0.97–5.42)	0.059	1.83 (1.05–3.19)	0.034
Metastases	3.32 (1.64–6.71)	0.001	2.57 (1.20–5.53)	0.016	3.26 (0.88–12.0)	0.076	2.80 (1.49–5.25)	0.001
Age (10yrs.)	1.59 (1.18–2.13)	0.002	1.59 (1.17–2.15)	0.003	1.60 (1.10–2.32)	0.015	1.56 (1.24–1.97)	<0.001
TU location								
Head	Ref.		-		-		-	
Body/Tail	0.61 (0.31–1.20)	0.155	-		-		-	
Multiple	1.50 (0.43–5.18)	0.526	-		-		-	
Symptoms								
None	Ref.							
Pain	1.79 (0.78–4.13)	0.172	1.89 (0.80–4.47)	0.146	1.40 (0.53–3.69)	0.493	1.58 (0.84–2.97)	0.154
Others	2.18 (1.03–4.62)	0.042	1.47 (0.66–3.28)	0.341	1.75 (0.53–5.81)	0.360	1.44 (0.76–2.73)	0.260

Tumor location was not included in the multivariable analysis due to multicollinearity. ASSO = Austrian Society of Surgical Oncology; CRP = C-reactive protein; HR = Hazards ratio; IMC = International multicenter; Ref. = Reference category; TU = pancreatic tumor; yrs = years.

**Table 3:** Patient characteristics, postoperative histopathological details, and outcomes according to pancreatic neuroendocrine neoplasm (panNEN) clinical risk score groups (combined cohort; *n* = 364).

	CRS: 0 (*n* = 82)	CRS: 1 (*n* = 157)	CRS: 2 (*n* = 103)	CRS: 3 (*n* = 22)	
**Risk score variables**	**Number (Percentage)**				
CRP ≥0.2 mg/dL	0 (0%)	112 (71.3%)	92 (89.3%)	22 (100%)	
Tumor size ≥3 cm	0 (0%)	43 (27.4%)	98 (95.1%)	22 (100%)	
Metastases present	0 (0%)	2 (1.3%)	16 (15.5%)	22 (100%)	
					
**Patient and tumor characteristics**	**Number (Percentage)**				***p***
Male gender	48 (58.5%)	88 (56.1%)	52 (50.5%)	13 (59.1%)	0.688
Age (years; median; IQR)	59.7 (15.0)	61.2 (16.8)	61.9 (16.1)	63.2 (18.6)	0.664
Grading (missing = 6)					<0.001
G1	55 (67.1%)	90 (58.1%)	37 (37.0%)	4 (19.0%)	
G2	26 (31.7%)	55 (35.5%)	46 (46.0%)	12 (57.1%)	
G3	1 (1.2%)	10 (6.5%)	17 (17.0%)	5 (23.8%)	
T3/T4 category	5 (6.1%)	32 (20.4%)	73 (70.9%)	19 (86.4%)	<0.001
Positive nodal status	16 (19.5%)	35 (22.3%)	34 (33.0%)	12 (54.5%)	0.002
R1 resection margin (missing = 3)	5 (6.2%)	13 (8.4%)	15 (14.6%)	3 (13.6%)	0.214
					
**Outcome**	**Number (Percentage)**				***p***
90-day overall complications	41 (50.0%)	83 (52.9%)	69 (67.0%)	18 (81.8%)	0.006
Pancreatic fistula ISGPF B/C	12 (14.6%)	21 (13.3%)	15 (14.3%)	3 (13.6%)	0.817
90-day severe complications	8 (9.8%)	15 (9.6%)	10 (9.7%)	4 (18.2%)	0.620
90-day mortality	1 (1.2%)	1 (0.6%)	5 (4.9%)	3 (13.6%)	0.004
5-year/10-year OS (estimated)	94.8%/94.8%	90.8%/74.0%	77.8%/51.4%	45.5%/34.1%	<0.001
5-year/10-year DSS (estimated)	98.0%/98.0%	97.3%/88.4%	85.7%/78.1%	52.7%/39.5%	<0.001
5-year/10-year RFS (estimated)	91.9%/84.2%	79.1%/66.3%	61.3%/48.8%	9.4%/0%	<0.001

CRP = C-reactive protein; CRS = clinical risk score; DSS = Disease-specific survival; IQR = Interquartile range; ISGPF = International study group for pancreatic fistula; OS = Overall survival; panNEN = pancreatic neuroendocrine neoplasm; RFS = Recurrence-free survival.

## Supplementary Materials

The following are available online at https://www.mdpi.com/2072-6694/12/5/1235/s1. Figure S1: Patient survival stratified by the Clinical Risk Score in the exploration and validation cohort; Table S1: Patient and Tumor Characteristics According to Preoperative C-Reactive Protein Values; Table S2: Association of Grading with Overall Survival; Table S3: Anonymized Patient Data; Supplement File: STROBE Checklist.
