# Supplementary material for: A Preoperative Clinical Risk Score Including C-Reactive Protein Predicts Histological Tumor Characteristics and Patient Survival after Surgery for Sporadic Non-Functional Pancreatic Neuroendocrine Neoplasms: An International Multicenter Cohort Study
Source: Cancers (Basel). 2020 May 14;12(5):1235. doi: 10.3390/cancers12051235 (PMC7280962; doi:10.3390/cancers12051235)
Supplement: Supplementary file 1 [file cancers-12-01235-s001.zip › Figure S1.docx]

**Supplemental Digital Content**


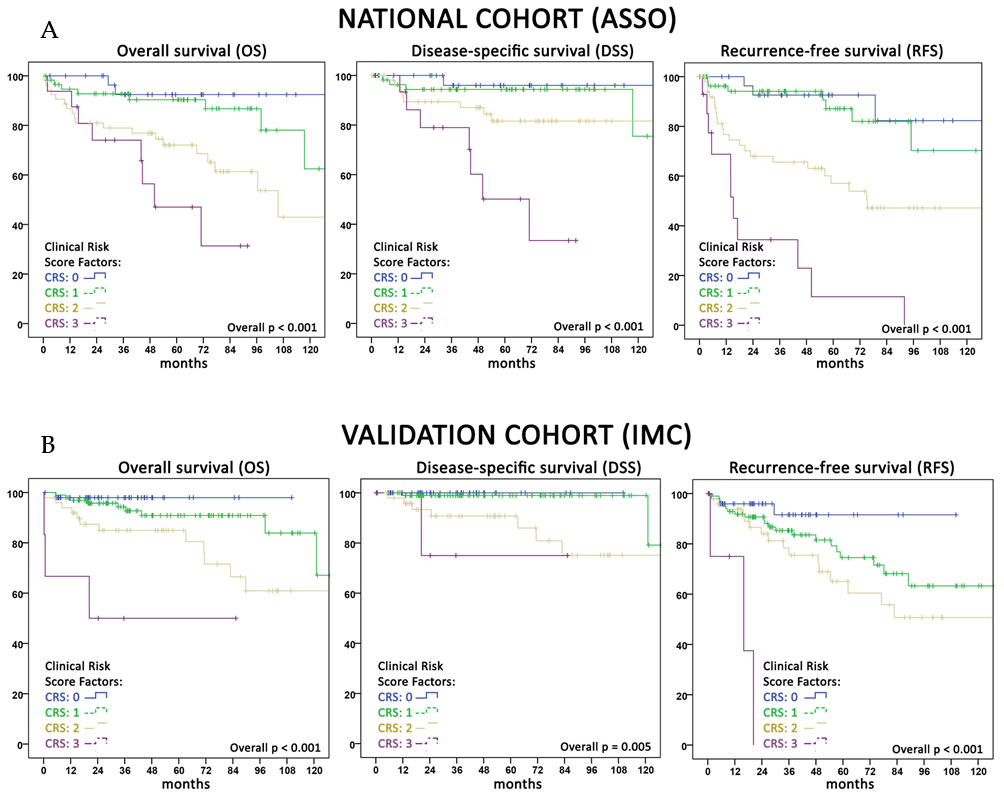


**Figure S1.** Patient survival stratified by the Clinical Risk Score in the exploration and validation cohort. A) Overall, Disease-specific and Recurrence-free Survival stratified by the Clinical Risk Score with 3 Factors (Metastases, CRP ≥0.2 mg/dL and Primary Tumor-size ≥3cm) in the National ASSO Cohort (Austrian Society for Surgical Oncology; *n* = 160). B) Overall, Disease-specific and Recurrence-free Survival stratified by the Clinical Risk Score with 3 Factors (Metastases, CRP ≥0.2 mg/dl and Primary Tumor-size ≥3cm) in the International Validation Cohort (*n* = 204).
