# Supplementary material for: A Preoperative Clinical Risk Score Including C-Reactive Protein Predicts Histological Tumor Characteristics and Patient Survival after Surgery for Sporadic Non-Functional Pancreatic Neuroendocrine Neoplasms: An International Multicenter Cohort Study
Source: Cancers (Basel). 2020 May 14;12(5):1235. doi: 10.3390/cancers12051235 (PMC7280962; doi:10.3390/cancers12051235)
Supplement: Supplementary file 1 [file cancers-12-01235-s001.zip › Table S1.docx]

**Supplemental Digital Content**

**Table S1.** Patient and Tumor Characteristics According to Preoperative C-Reactive Protein Values.

| **Variable** | **CRP <0.2 mg/dL (*n* = 138)** | **CRP** **≥0.2 mg/dL (*n* = 226)** |  |
| --- | --- | --- | --- |
|  | **Number (Percentage)** | **Number (Percentage)** | ***P*** |
| Female gender | 60 (43.5%) | 103 (45.6%) | 0.696 |
| Age (years; median; IQR) | 58.7 (51.3; 66.0) | 62.8 (52.9; 69.33) | 0.021 |
| Tumor location |  |  | 0.834 |
| Head | 54 (39.1%) | 82 (36.3%) |  |
| Body/Tail | 80 (58.0%) | 136 (60.2%) |  |
| Multiple | 4 (2.9%) | 8 (3.5%) |  |
| Preoperative symptoms |  |  | 0.047 |
| No symptoms | 96 (69.6%) | 143 (63.3%) |  |
| Pain | 28 (20.3%) | 38 (16.8%) |  |
| Others (e.g., jaundice) or multiple | 14 (10.1%) | 45 (19.9%) |  |
| Preoperative bilirubin ≥4 mg/dL | 5 (3.6%) | 12 (5.3%) | 0.459 |
| Tumor grade (missing: 6) |  |  | 0.048 |
| G1 | 74 (54.4%) | 112 (50.5%) |  |
| G2 | 56 (41.2%) | 83 (37.4%) |  |
| G3 | 6 (4.4%) | 27 (12.2%) |  |
| Tumor size ≥ 3cm | 54 (39.1%) | 109 (48.2%) | 0.090 |
| ENETS T stage |  |  | 0.182 |
| T1/2 | 95 (68.8%) | 140 (61.9%) |  |
| T3/4 | 43 (31.2%) | 86 (38.1%) |  |
| Nodal status |  |  | 0.501 |
| Nodal-negative | 82 (59.4%) | 134 (59.3%) |  |
| Nodal-positive | 40 (29.0%) | 57 (25.2%) |  |
| Nodal-status unknown (Nx) | 16 (11.6%) | 35 (15.5%) |  |
| Metastases present | 13 (9.4%) | 27 (11.9%) | 0.455 |
| R0 Resection margin (missing: 3) | 125 (91.9%) | 200 (88.9%) | 0.353 |
| 90-day severe morbidity | 13 (9.4%) | 24 (10.6%) | 0.713 |
| 90-day mortality | 1 (0.7%) | 9 (4.0%) | 0.097 |
| Follow-up for OS (months; median; IQR) | 38.9 (20.3; 65.7) | 54.0 (24.6; 87.3) | 0.006 |
| Death during follow-up | 7 (5.1%) | 57 (25.2%) | <0.001 |
| Follow-up for DFS (months; median; IQR) | 33.0 (18.7; 56.0) | 38.3 (14.0; 78.2) | 0.140 |
| Recurrence during follow-up (missing: 4) | 29 (21.2%) | 57 (25.6%) | 0.343 |

CRP = C-reactive protein; DFS = Disease-free survival; ENETS = European neuroendocrine tumor society; IQR = Interquartile range; OS = Overall survival; yr = year
