# Supplementary material for: A Preoperative Clinical Risk Score Including C-Reactive Protein Predicts Histological Tumor Characteristics and Patient Survival after Surgery for Sporadic Non-Functional Pancreatic Neuroendocrine Neoplasms: An International Multicenter Cohort Study
Source: Cancers (Basel). 2020 May 14;12(5):1235. doi: 10.3390/cancers12051235 (PMC7280962; doi:10.3390/cancers12051235)
Supplement: Supplementary file 1 [file cancers-12-01235-s001.zip › Table S2.docx]

**Supplemental Digital Content**

**Table S2.** Association of Grading with Overall Survival.

| **A) Overall Survival Curve according to Grading (G1 vs. G2/G3)* in the whole cohort (*n* = 358; grading missing = 6)** | **B) Multivariable Analysis of Preoperative Factors including Grading associated with Overall Survival** | | |
| --- | --- | --- | --- |
| 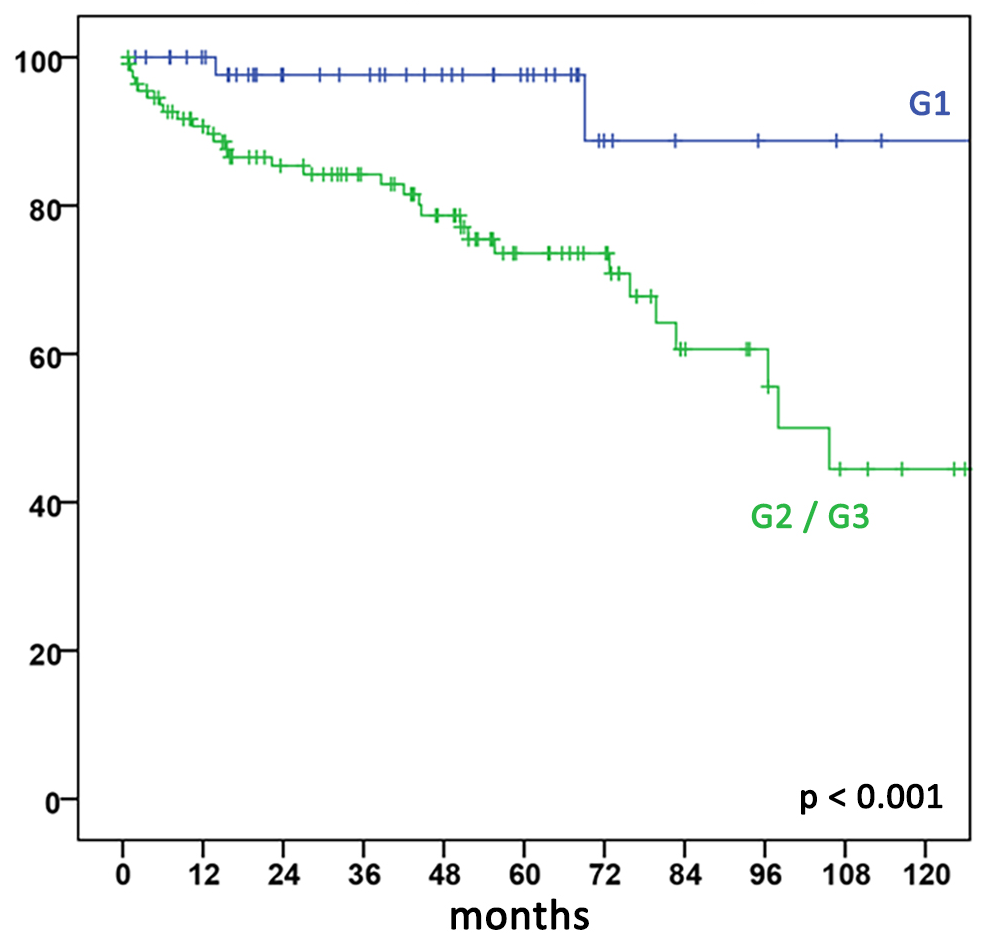 |  | **Multivariable Analysis** | |
|  | **Factor** | **Combined (*n* = 358)** | |
|  |  | **HR (95%CI)** | ***P*** |
|  | Male sex | 1.02 (0.61-1.70) | 0.938 |
|  | CRP ≥0.2 mg/dL | 3.85 (1.64-9.05) | 0.002 |
|  | TU size ≥3cm | 1.68 (0.95-2.97) | 0.073 |
|  | Metastases | 2.02 (1.05-3.87) | 0.035 |
|  | Age (per 10yrs.) | 1.56 (1.24-1.98) | <0.001 |
|  | Symptoms |  |  |
|  | None |  |  |
|  | Pain | 1.58 (0.84-2.97) | 0.154 |
|  | Others | 1.44 (0.76-2.73) | 0.260 |
|  | Grading G2/G3 vs. G1* | 2.09 (1.17-3.74) | 0.013 |
|  | * Due to limited availability of preoperative biopsy results WHO grading was assessed in surgical specimen and the result was used as a surrogate parameter for preoperative biopsy grading.  ASSO = Austrian Society of Surgical Oncology; CRP = C-reactive protein; HR = Hazards ratio; IMC = International multicenter; Ref. = Reference category; TU = pancreatic tumor; yrs = years. | | |
